# Machine Learning for Predicting Mechanical Properties of 3D-Printed Polymers from Process Parameters: A Review

**DOI:** 10.3390/polym18040499

**Published:** 2026-02-17

**Authors:** Savvas Koltsakidis, Emmanouil K. Tzimtzimis, Dimitrios Tzetzis

**Affiliations:** Digital Manufacturing and Materials Characterization Laboratory, School of Science and Technology, International Hellenic University, 57001 Thessaloniki, Greece; skoltsakidis@ihu.edu.gr (S.K.); m.tzimtzimis@ihu.edu.gr (E.K.T.)

**Keywords:** additive manufacturing, machine learning, artificial neural networks, mechanical properties, process parameters

## Abstract

Polymer additive manufacturing (AM) has grown rapidly in the past decade, with material extrusion, vat photopolymerization, powder bed fusion and jetting now widely used for functional polymer parts. The mechanical performance of these parts depends strongly on process parameters such as layer height, build orientation, energy input and post-processing conditions, which motivate the development of predictive models for process–property relationships. Classical approaches based on Taguchi designs, ANOVA and response surface methodology have provided valuable insight, but the potential of modern machine learning (ML) techniques is not yet fully exploited. This review surveys recent work on ML-based prediction of mechanical properties of polymer AM parts using process parameters as inputs. Across the literature, well-tuned artificial neural networks, tree-based ensembles and support vector regression typically achieve prediction errors below about 5–10% for strength and modulus, showing that data-driven surrogates can substantially reduce experimental trial-and-error in process optimization. Ongoing challenges include small datasets, missing standardized error metrics, and limited coverage of non-quasi-static phenomena like fatigue, impact, and environmental degradation.

## 1. Introduction

Additive manufacturing (AM) has evolved from a rapid prototyping tool into a key manufacturing technology across aerospace, automotive, biomedical and consumer sectors. By building components layer by layer directly from digital models, AM enables complex geometries, mass customization and rapid design iteration that are difficult or impossible to achieve with conventional subtractive or formative processes [1,2,3,4]. In economic terms, polymer-based AM already accounts for a large fraction of installed systems and printed parts, with steady growth projected over the coming decade in both high-value and commodity applications [1,3,5].

Within this broader landscape, polymer additive manufacturing has seen particularly rapid development in terms of materials, hardware and applications. Material extrusion, vat photopolymerization, powder bed fusion and jetting processes now cover a wide range of thermoplastics, elastomers and photopolymers, including multi-material and functionally graded systems [5,6,7,8,9] as well as hierarchical porosity [10,11,12,13,14]. Recent reviews emphasize how advances in polymer chemistry, rheology control and machine design have expanded the accessible property space from rigid structural polymers to flexible, conductive and stimuli responsive materials, enabling applications in soft robotics, electronics, tissue engineering and lightweight structures [6,7,8,9].

Across these technologies, the mechanical performance of printed parts depends sensitively on process parameters that control heat input, curing, cooling and material deposition. In fused filament fabrication and related extrusion processes, variables such as nozzle and bed temperature, layer height, infill density, print speed and build orientation strongly influence porosity, interlayer bonding and crystallinity, with direct consequences for tensile and impact properties [15,16,17]. Comparable sensitivities exist in vat photopolymerization, where exposure time, layer thickness, resin formulation and post-curing determine crosslink density and defect content [8,9], and in polymer powder bed fusion, where laser power, scan speed, hatch spacing and bed temperature govern the density and morphology of sintered PA12 and related powders [1,18]. As a result, nominally identical materials processed on different machines or with different parameter sets can exhibit markedly different stiffness, strength and ductility [9,18,19,20].

Given this strong process sensitivity, reliable prediction and control of mechanical properties have become central challenges for polymer AM. Mechanical behavior is often anisotropic, with properties depending on build orientation and internal architecture, and can be affected by defects at multiple length scales. Traditionally, these process–property relationships have been explored using classical designs of experiments, regression analysis and mechanistic models. Taguchi methods, response surface methodology and analysis of variance have been widely used to identify influential parameters and to build low-order polynomial models for responses such as tensile strength or surface roughness, particularly in fused filament fabrication [15,16,17].

In parallel, machine learning has emerged as a powerful tool for extracting patterns from experimental and simulation data in additive manufacturing. Recent reviews document applications of supervised and unsupervised learning across the AM workflow, including design for AM, topology optimization, process monitoring, defect detection, microstructure prediction and build-time estimation [21,22,23,24]. For example, in metals, deep learning and other data-driven models are increasingly used to link process parameters and in situ sensor signals to melt pool behavior, porosity and resulting mechanical properties [23,24,25,26]. In addition, machine learning has been utilized for optimizing functionally graded materials [27].

The present review focuses specifically on machine learning-based prediction of mechanical properties of polymer additively manufactured parts using process parameters as inputs. First, the relevant mechanical behavior of common polymer materials is summarized and the main polymer AM technologies, emphasizing the role of process parameters in shaping microstructure and anisotropy. Following that, a survey is conducted on the published work on machine learning models that map process settings to mechanical properties for material extrusion, vat photopolymerization, polymer powder bed fusion and jetting, comparing model choices, dataset design and reported accuracy. Building on this analysis, the current limitations, including gaps in property coverage, dataset size and standardization, are discussed and promising future directions are highlighted. The goal is to provide a structured picture of the state of the field and to identify opportunities where machine learning can most effectively support the reliable mechanical design of polymer 3D-printed components.

## 2. Fundamentals

### 2.1. The Role of Process Parameters in Polymer 3D Printing

**Figure 1:**
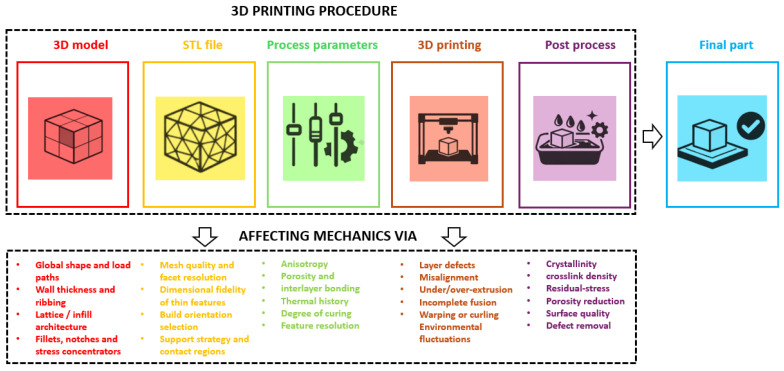
Schematic workflow of polymer 3D printing, from digital design to final part. Starting from a 3D model, the geometry is converted to a mesh file, processed in slicing software to generate toolpaths and process parameters, fabricated layer-by-layer on the printer and then subjected to post-processing before the final component is obtained (icons without text labels).

Polymer additive manufacturing is a fully digital workflow that links virtual design to physical parts. As presented in Figure 1, a 3D model is first created, exported to a mesh file (typically STL), processed in slicing software to generate layers and toolpaths, and then fabricated layer-by-layer on the printer. Post-processing steps such as support removal, cleaning, thermal treatment or UV curing complete the workflow and can significantly modify the final properties [3,4,28]. At each stage, user-defined settings determine the local thermal and mechanical history of the material and therefore the structure and properties of the printed component. Mesh quality affects the accuracy of the toolpath, process parameters control the local thermal history, porosity, anisotropy and surface resolution, and post-processing modifies crystallinity, crosslinking and residual stresses. This review focuses on the effect of process parameters.

**Figure 2:**
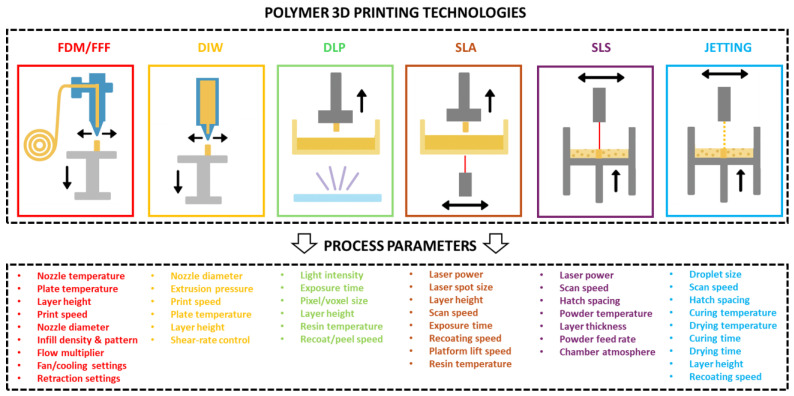
Schematic overview of polymer additive manufacturing methods. The figure groups processes into material extrusion (FDM/FFF, DIW), vat photopolymerization (SLA, DLP, CLIP), powder bed fusion (polymer SLS and related processes), material jetting and binder jetting. The process parameters for each method are presented.

Polymer AM processes fall mainly into five families: material extrusion, vat photopolymerization, powder bed fusion, material jetting and binder jetting [3,4,18,28,29]. Figure 2 schematically illustrates these methods. Material extrusion covers fused deposition modeling/fused filament fabrication (FDM/FFF) and direct ink writing (DIW); vat photopolymerization includes stereolithography (SLA), digital light processing (DLP) and related techniques; powder bed fusion is represented primarily by polymer selective laser sintering (SLS) and Multi Jet Fusion; material jetting corresponds to PolyJet-type processes; and binder jetting uses a liquid binder to selectively join a polymer or composite powder bed [18,28,29].

For each technology, the process parameters control how the material is deposited, fused and solidified, thereby dictating porosity, crystallinity, residual stresses and ultimately mechanical performance. In material extrusion, key parameters include the nozzle and bed temperature, layer height, extrusion rate, print speed, raster width and angle, infill density and pattern, cooling conditions and build orientation. Experiments on FDM/FFF parts show that higher infill density, suitable nozzle temperature and raster alignment with the load direction generally increase the tensile and flexural strength, whereas coarse layer heights, low extrusion temperatures or unfavorable raster angles promote void formation and weak interlayer bonding, leading to strong anisotropy and reduced strength [30,31,32]. DIW shares similar geometric parameters (layer height, path spacing, build orientation) but is additionally controlled by ink rheology and dispensing pressure, which govern filament stability and porosity in printed lattices and cellular structures [29].

In vat photopolymerization, the main parameters are layer thickness, exposure time or energy, light intensity, scanning speed, peel or lift speed, resin temperature and post-curing conditions. These variables determine cure depth, degree of conversion and crosslink density. Studies on SLA and DLP resins show that increasing exposure or post-cure time typically raises the modulus and strength up to a saturation point, after which over-curing can embrittle the material or degrade accuracy [29,33]. Layer thickness and exposure are coupled: thin layers improve vertical resolution but require sufficient energy to ensure interlayer bonding, while thicker layers can reduce build time but increase staircase effects and internal stresses [29,33]. Part orientation influences the direction of layer stacking relative to applied loads and the amount of support contact, so tensile properties often differ between flat, edge and vertical orientations, although anisotropy is usually less pronounced than in FDM when curing is optimized [29,33].

Polymer powder bed fusion processes such as SLS depend primarily on laser power, scan speed, hatch spacing, scan strategy, layer thickness, bed temperature and part placement. These can be condensed into a volumetric energy density, which correlates strongly with the density, crystallinity and mechanical properties of PA12 and related powders [18]. Too little energy leads to incomplete sintering, high porosity and low tensile strength; excessive energy can cause degradation, warping and poor surface finish. Maintaining the powder bed just below the melting temperature promotes good neck growth between particles and reduces residual stresses, improving ductility and dimensional stability [18]. Build orientation and location within the bed influence local cooling rates and thus crystallinity, which again affects modulus and elongation [18].

In material jetting, typical parameters include droplet size and spacing, layer thickness, printhead temperature, UV exposure per layer, surface-finish mode and build orientation, as well as the mixing ratio in multi-material “digital” polymers. These settings control how droplets coalesce and cure, which in turn affects porosity, interlayer adhesion and microstructural gradients. Experimental studies report that the orientation with respect to the printhead direction and the choice of matte versus glossy mode can change the tensile and flexural strength and surface hardness, while the spatial distribution of rigid and flexible components in digital materials tunes the effective modulus and failure strain [34].

Binder jetting separates shaping from densification. During printing, layer thickness, binder saturation, droplet size, recoating speed and powder characteristics govern the density and cohesion of the green body. The subsequent curing, debinding and sintering steps define the final porosity and mechanical properties. Reviews show that higher binder saturation and thinner layers tend to increase green strength and reduce porosity, but may worsen the dimensional accuracy; sintering temperature and time then determine the trade-off between densification, shrinkage and strength [35]. For polymer powders and polymer–binder systems, the interplay between the powder morphology, binder chemistry and thermal cycle is critical for achieving adequate stiffness and toughness [35].

### 2.2. Effect of Process Parameters to Mechanical Properties

The mechanical response of polymers is central to the performance of 3D-printed parts and to the choice of suitable targets for ML models. Polymeric materials show strong temperature and strain-rate dependence, pronounced viscoelasticity and a broad spectrum of behaviors, from brittle glassy solids to highly extensible elastomers. These properties are governed by the molecular architecture, degree of crystallinity, entanglement density and the presence of fillers or reinforcements, as described in standard polymer mechanics texts [36,37,38,39].

In polymer additive manufacturing (AM), the primary mechanical descriptors are tensile (Young’s) modulus, yield stress, ultimate tensile strength, elongation at break, flexural modulus and strength, impact resistance and fracture toughness which are typically obtained from uniaxial tensile tests. For common engineering thermoplastics, tensile moduli typically lie between about 1 and 4 GPa, tensile strengths between roughly 30 and 100 MPa, and elongation at break can range from a few percent for brittle, glassy materials to several hundred percent for elastomers and flexible thermoplastic polyurethanes [18,35,40,41,42,43].

From an AM perspective, process parameters determine how far the effective properties of a printed part depart from these bulk values. In FDM/FFF, layer height, infill density, infill pattern, raster angle, print speed and build orientation all have a strong influence on tensile and flexural properties. Taguchi and factorial studies for PLA and ABS consistently show that higher infill density and optimized infill patterns (for example, hexagonal or gyroid) markedly increase stiffness and strength, while oblique raster angles (such as ±45 degrees) and “on-edge” or vertical build orientations significantly improve the load-bearing capacity in the loading direction [35,41,44,45].

In polymer SLS and related powder bed fusion processes, the key parameters are laser power, scan speed, hatch spacing, layer thickness, preheating or bed temperature and part placement. These can be combined into an energy density measure that correlates strongly with part density, tensile strength, modulus and elongation at break [46,47]. Insufficient energy density leads to high porosity, reduced strength and brittle behavior, whereas overly high energy density can cause thermal degradation, warping and dimensional inaccuracy. Preheating close to but below the melting point improves interlayer bonding and ductility, while build orientation and part position in the bed introduce additional anisotropy and variability in tensile and flexural properties [46,47].

In vat photopolymerization, the response of crosslinked resins depends strongly on exposure conditions and post-processing. Layer thickness, exposure time or energy, build orientation and support strategy all affect cure depth, crosslink density and defect distribution, which in turn control modulus, strength and elongation [48,49,50]. UV post-curing can increase tensile strength and stiffness relative to the as-printed state, by driving the conversion of residual monomers and unreacted oligomers. Orientation effects can be significant: specimens printed vertically or at oblique angles may show lower strength and higher scatter than those printed flat, because of the differences in layer stacking and support contact regions [48,49,50].

In material and binder jetting processes, parameters such as layer thickness, tray location, build orientation, print mode (for example, high-quality versus high-speed), surface finish (matte versus glossy), material mixing ratio and post-curing or infiltration all influence mechanical properties [37,51,52]. For PolyJet and MultiJet systems, different raster orientations and surface finishes show substantial changes in tensile and flexural strength, as well as in dimensional accuracy and surface hardness, depending on build mode and part placement across the tray. In multi-material jetting, digital materials formed by mixing rigid and rubber-like photopolymers allow tailoring of the modulus and toughness but also introduce additional process–composition interactions that affect anisotropy and long-term stability [37,51,52].

### 2.3. Machine Learning Models

In this review, machine learning is used in a supervised way, where the goal is to learn a function that maps a set of inputs, such as process parameters and material descriptors, to one or more outputs, such as tensile strength, Young’s modulus or elongation at break. The only information the algorithm receives is a dataset that contains the input process parameters and the output measured mechanical properties. During training, the model adjusts its internal parameters so that its predictions are as close as possible to the measured values, usually by minimizing a loss function like the mean squared error on the training set [53,54,55]. To check if the model has really learned general trends rather than simply memorizing the training data, the available dataset is split into training, validation and test sets, and procedures such as k-fold cross-validation are used [53,54,55,56].

**Figure 3:**
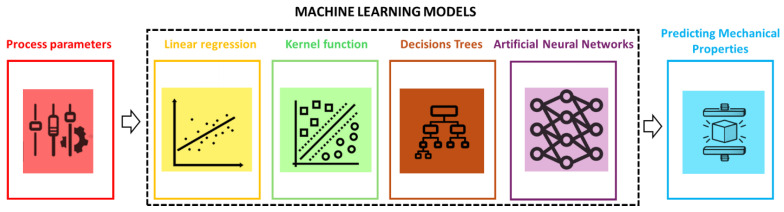
Schematic representation of the machine learning workflow used in this review. Process parameters (**left**) are provided as inputs to different supervised learning models (linear regression, kernel-based methods, decision tree ensembles and artificial neural networks), which act as surrogates to map process settings to target responses. The predicted outputs are mechanical properties of the printed part, illustrated here by a cube under compression (**right**).

Figure 3 schematically summarizes the main model families that appear in the polymer AM literature for mechanical properties predictions. Linear regression assumes that the target property can be written as a weighted sum of the inputs plus a constant term. The weights and bias are chosen so that the squared difference between predicted and measured values is as small as possible. Regularized variants such as ridge and lasso regression add a penalty on large weights in order to discourage overfitting and make the model more stable [53,55]. These models are easy to understand and interpret and they often perform surprisingly well when the response varies approximately linearly over the explored range of process parameters, which is sometimes the case for modest changes in the layer height, infill or exposure time [57,58].

Kernel methods, in particular support vector regression, extend linear regression by allowing the model to represent curved relationships. Instead of fitting a straight line in the original feature space, they fit a linear model in a higher dimensional feature space that is accessed indirectly through a kernel function, for example, a radial basis function that measures how similar two parameter sets are [53,54]. Support vector regression aims to find a smooth function that passes as close as possible to the data points while keeping most of them within a specified error band. With suitable choices of kernel and regularization, these methods can capture nonlinear process–property relationships using relatively small datasets, which is attractive for additive manufacturing where full mechanical test campaigns often provide only tens of data points [58,59].

Decision trees follow a different idea. They split the input space into regions by asking a sequence of simple questions on individual features, such as “is infill density larger than 60 percent” or “is energy density above a threshold”. Each leaf of the tree corresponds to a region with an approximately constant prediction, for example, the average tensile strength of the samples that fall into that region [53,56]. A single tree is intuitive but often unstable. In polymer AM, most studies therefore use ensembles of trees. Random Forests build many trees on different random subsets of the data and average their predictions, which reduces sensitivity to noise and overfitting. Gradient boosting builds trees one after another, where each new tree tries to correct the errors made by the current ensemble. Reviews and benchmarking studies in additive manufacturing show that these ensemble methods usually provide an excellent balance between accuracy, robustness and computational cost for tabular process–property data [57,58,59,60]. They also offer useful interpretability, because measures of feature importance and tools such as SHAP values can highlight which process parameters have the largest effect on the predicted properties, for example, infill density and build orientation in FDM or energy density and bed temperature in SLS [58,59,60].

Artificial neural networks approximate complex nonlinear relationships by stacking many simple computational units, called neurons, into layers. Each neuron computes a weighted sum of its inputs, adds a bias and then applies a nonlinear activation function. By composing several layers, the network can gradually transform raw inputs, for example, numerical process parameters, into higher level internal representations that are useful for predicting mechanical properties. The weights and biases are learned by minimizing a loss function on the training data using gradient-based optimization and backpropagation [60,61]. In polymer additive manufacturing, most studies use relatively small fully connected networks due to limited dataset sizes, but even these modest architectures are able to capture strong nonlinearities and interactions between process parameters. Reported errors for tensile strength and modulus often lie in the range of about 5 to 10 percent when the experimental design covers the relevant parameter space and appropriate regularization is used [58,59,60,61]. When additional inputs such as images from in situ monitoring or micro computed tomography are available, convolutional neural networks are employed so that the model can learn spatial features like porosity patterns or lack of fusion defects [57,61].

Across all these model families, the workflow has several common steps. Input features are usually scaled so that parameters with different units, for example, temperature and speed, have comparable numerical ranges. Hyperparameters, such as the strength of regularization in linear models, the kernel width and error tolerance in support vector regression, the number and depth of trees in ensembles, or the number of layers and neurons in neural networks, are chosen by systematic search combined with cross-validation [53,54,55,56]. Model performance is then reported on a held out test set using metrics such as the mean squared error, root mean squared error, mean absolute error and the coefficient of determination $R^{2}$. In order to compare performance across different mechanical properties, which can have very different magnitudes and units, normalized metrics such as mean absolute percentage error are also widely used [58,59,60,61,62].

## 3. Machine Learning-Based Prediction of Mechanical Properties from Process Parameters

This section reviews how machine learning (ML) has been used to model the relationship between the process parameters and mechanical properties of 3D-printed polymers. Firstly, a focus on extrusion-based technologies is presented, where most of the work has been carried out. Subsequent subsections will extend the discussion to other polymer AM technologies.

### 3.1. Machine Learning-Based Prediction of Mechanical Properties in Extrusion Processes

FDM/FFF is the most extensively studied polymer AM process in terms of ML-based property prediction, mainly due its accessibility and because part strength and stiffness depend sensitively on parameters such as layer height, raster orientation, infill density, pattern, and extrusion temperature. Early works concentrated on neat PLA and showed that neural networks (ANNs) can reliably predict tensile strength from a small set of process variables. Pazhamannil et al. trained a feed-forward ANN on a Taguchi L9 dataset to map nozzle temperature, layer thickness and infill speed to the ultimate tensile strength of PLA specimens; their model reproduced confirmation experiments within about 5% error, demonstrating that data-driven surrogates can capture key nonlinearities in FDM processing with limited experimental effort [63]. Vendittoli et al. later used Bayesian-regularized ANNs with seven input parameters (including layer height, printing speed, extrusion and bed temperatures, and infill settings) to predict the tensile strength of PLA parts printed under a Taguchi L16 design, achieving very low mean errors and correlation coefficients close to unity on validation data [64]. Both studies rely on relatively small design-of-experiments datasets, with nine and 16 unique parameter combinations respectively, and use either confirmation experiments or simple train–test splits.

**Figure 4:**
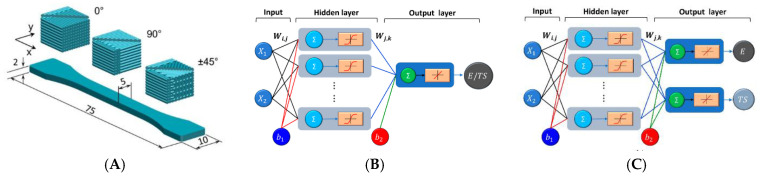
(**A**) Geometry of the 3D-printed samples and printing direction (all dimensions in mm). (**B**) Configuration with one output (Young’s modulus or tensile strength). (**C**) Configuration with two outputs (Young’s modulus and tensile strength) [65].

Moving beyond PLA, several studies have employed ANNs to predict the mechanical properties of engineering thermoplastics and fiber-reinforced composites produced by FDM/FFF. Fetecau et al. modeled the Young’s modulus and tensile strength of PA12 and short fiber-reinforced PA12 composites from only two parameters, printing orientation and infill density (Figure 4A), using single- and multi-output ANN architectures (Figure 4B,C) showing relative prediction errors generally below 5%, with infill density and 0° raster orientation identified as the dominant factors controlling stiffness and strength [65]. Ulkir et al. investigated carbon fiber-reinforced PA12 (PA12-CF) and developed an ANN model that simultaneously predicts tensile strength and surface roughness from five FDM parameters (layer thickness, infill pattern, nozzle temperature, printing speed and infill density). Designed via a Taguchi L27 matrix, their experiments showed that infill density is the main contributor to tensile strength, whereas layer thickness governs surface roughness; the trained ANN achieved $R^{2}>0.99$ and mean validation errors below 0.5% [66]. Similar ANN-based frameworks have been reported for flexural properties of carbon fiber-reinforced polyamide parts, where response surface methodology (RSM) and ANN models both capture the strong influence of raster angle and build-plate temperature on flexural modulus and strength [67]. Taken together, these PA12-based studies typically use on the order of 20–30 distinct process parameter combinations and shallow feed-forward networks yet still report relative errors in the few-percent range.

In parallel, ensemble and tree-based methods have been explored as competitive alternatives to ANNs for FDM/FFF property prediction. Deb et al. compared multiple ensemble regressors, Random Forest, Gradient Boosting, XGBoost, AdaBoost and Extremely Randomized Trees, for predicting the tensile strength and surface roughness of PLA specimens as functions of build orientation, infill angle, layer thickness, printing speed and nozzle temperature in a Taguchi L27 design [68]. Ensemble models substantially outperformed linear and kernel-based baselines, with Extremely Randomized Trees giving mean absolute percentage errors around 2–3% for tensile strength and Random Forest performing best for surface roughness. Özkül et al. performed an extensive benchmark of 25 ML algorithms (including tree ensembles, support vector regression and k-nearest neighbors) to predict hardness, tensile strength and flexural strength of FDM-produced polymer parts. Their results confirmed that gradient-boosted trees and optimized Random Forest models offer an excellent compromise between accuracy, robustness and training cost across different mechanical targets and parameter combinations [69]. In both works, the effective dataset size remains relatively small and validation is carried out via simple train–test splits or repeated random splitting rather than systematic k-fold cross-validation.

A complementary research direction incorporates in situ sensing into the input space of ML models to improve robustness and enable more physics-aware predictions. Liu et al. equipped an FFF system with force and temperature sensors near the nozzle and used several ML regressors, including support vector regression and ensemble methods, to predict the extrusion force and mechanical properties from both process parameters and sensor features [70]. Compared with purely statistical response surface models, ML approaches achieved higher coefficients of determination and better generalization, highlighting the value of combining process settings with real-time process signatures. This study also illustrates that, once sensor information is included, acceptable prediction accuracy can be obtained with relatively small numbers of printed specimens, provided that inputs and outputs are carefully curated.

**Figure 5:**
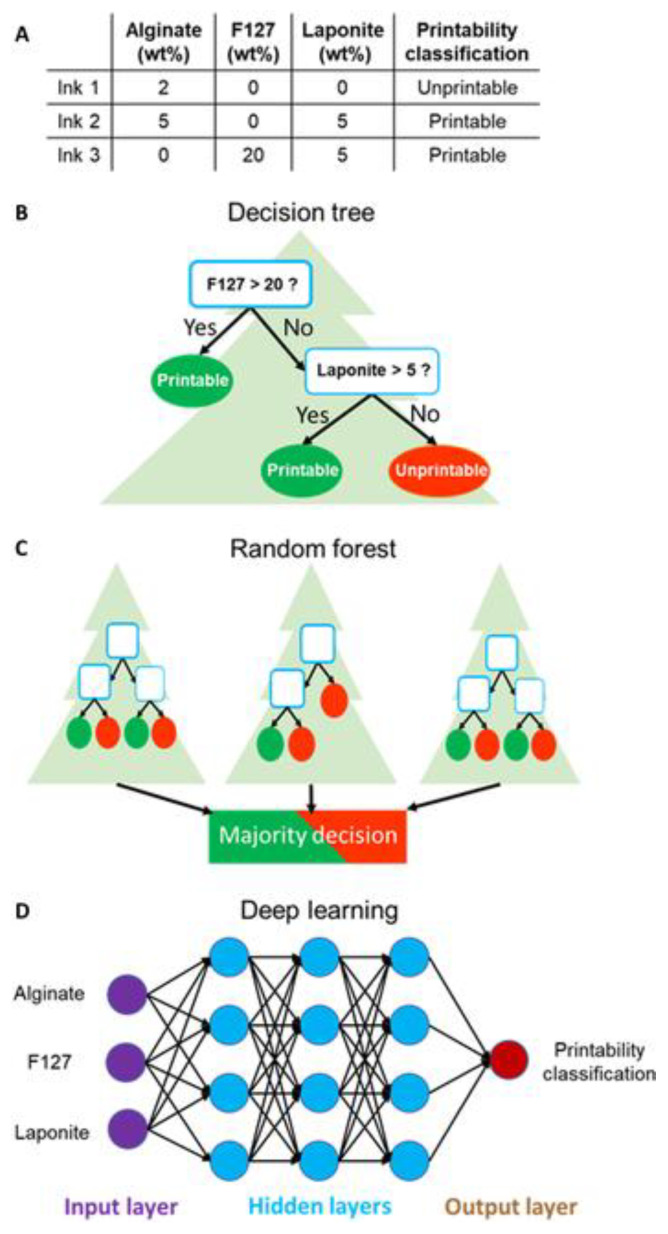
The working principles of the machine learning algorithms. (**A**) Example of a dataset of printability of hydrogel formulations. Each item (ink) is associated with several features (hydrogel type/name) and one output (printability). Schematics of the internal structures of machine learning algorithms include (**B**) decision tree, (**C**) Random Forest, and (**D**) deep learning after learning from the dataset [72].

For robocasting/direct ink writing (DIW), the use of ML for mechanical property prediction is more recent but shows similar trends to FDM/FFF. Roach et al. used computer-vision features extracted from single cross-sectional images of DIW-printed foam replacement structures as inputs to an artificial neural network, accurately predicting their full compression stress–strain response (including elastic modulus, plateau stress and densification strain) and then coupling the model with a genetic algorithm to identify filament diameters and spacings that achieve a target compression curve [71]. Here, the dataset consisted of several hundred printed and imaged lattice variants, and the authors explicitly adopted a train/validation/test split, which contrasts with the single hold-out strategies more common in early FFF work. In the bioprinting domain, Chen et al. trained decision tree, Random Forest and deep learning models to map biomaterial formulation and rheological descriptors to DIW printability and shape fidelity, providing an indirect way to control the structural integrity and mechanical stability of printed hydrogel constructs via ink design rather than purely geometric tuning [72]. Figure 5 presents an example of a dataset of the printability of hydrogel formulations that was utilized where each item (ink) is associated with several features (hydrogel type/name) and one output (printability). Although the primary output in this case is printability rather than a standard mechanical test, the study demonstrates how larger formulation datasets (a total of 210 formulations) can be used to build more generalizable models, in contrast to the much smaller Taguchi-based datasets.

### 3.2. Machine Learning-Based Prediction of Mechanical Properties in Vat Photopolymerization

Vat photopolymerization (VP) processes such as stereolithography (SLA) and digital light processing (DLP) are increasingly combined with ML models to relate exposure conditions, build orientation, resin formulation and post-curing to the mechanical properties of cured photopolymers. Compared with extrusion-based methods, VP studies place more emphasis on curing-related parameters (light intensity, exposure time, layer thickness) and on architected structures, but the goal is similar: build predictive surrogates that can guide process windows and structural design for target stiffness, strength and toughness [73]. In most cases the available datasets remain relatively modest, typically a few dozen parameter combinations for bulk coupons and on the order of tens to low hundreds of samples for lattices, and authors use simple train–test splits or hold-out validation rather than systematic k-fold cross-validation.

A first group of works focuses on parameter–property regression for bulk-like VP parts. Kadauw developed four artificial neural network (ANN) models to predict tensile strength, yield strength, Shore D hardness and surface roughness of an ABS-like photopolymer printed by SLA, using only four process parameters as inputs: build orientation, lifting speed, lifting distance and exposure time [74]. Based on 54 experimental builds and subsequent mechanical testing, the ANNs achieved correlation coefficients of ~0.98 for all three mechanical properties and roughness, and were then used in a multi-objective optimization step to identify an orientation/speed/exposure combination that simultaneously maximizes strength and hardness while minimizing Ra.

For DLP-printed polymers, Kavimani et al. examined the influence of light intensity, part orientation and exposure time on the tensile, flexural and impact properties of PLA specimens produced by DLP and trained an ANN to map these three process variables to the three mechanical responses [75]. Their experiments showed that light intensity is the dominant factor for tensile and flexural strength, while impact strength is primarily controlled by exposure time [75]. The trained ANN predicted the mechanical properties with small deviations from the experimental values and outperformed linear regression, after which a multi-criteria optimization identified the optimal combination of light intensity, orientation and exposure. Here again the dataset is comprised from a Taguchi L9 matrix, yet the reported R values for the developed ANN model are higher than 0.99.

A second line of work targets architected photopolymer lattices produced by VP, where ML models take both geometry and process-related factors as inputs. Uddin and Fan proposed a ML framework for photopolymer gyroid lattices fabricated by commercial vat photopolymerization printers, predicting Young’s modulus, yield strength, fracture strength, fracture strain and toughness across several porosities and strain rates [76]. In this case, the authors explicitly report using a train/test split combined with grid-search hyperparameter tuning. They compared Random Forest, Extremely Randomized Trees, feed-forward ANN and LSTM models, finding that tree ensembles provided the best accuracy at low computational cost. SHAP analysis confirmed that porosity and strain rate are the main drivers for the predicted properties, and the framework offers a compact surrogate for exploring energy-absorbing lattice designs without exhaustive mechanical testing.

**Figure 6:**
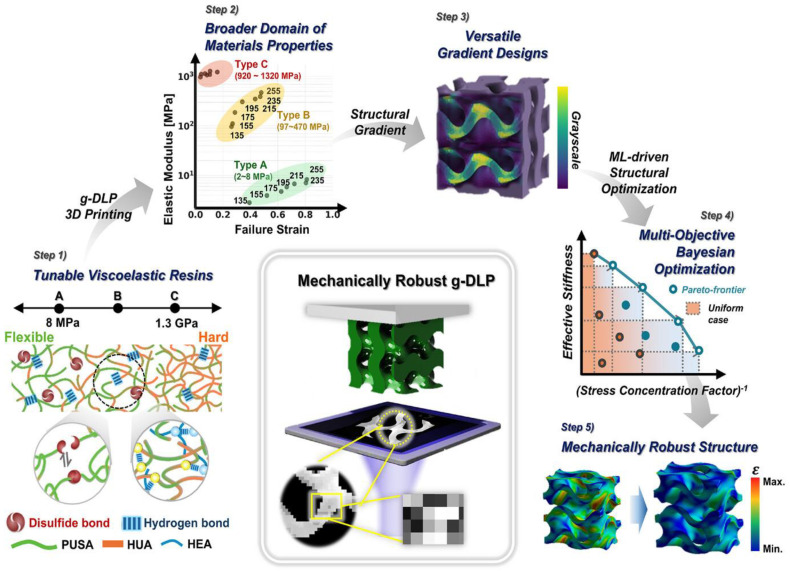
Overview of the material-to-structure design and fabrication strategies for creating mechanically robust g-DLP printed structures. Step 1: Development of highly tunable PUA with dynamic bonds to enhance viscoelastic damping. Step 2: g-DLP 3D printing of viscoelastic PUSA-HUA resins to achieve an expanded range of mechanical properties. Step 3: Implementation of a versatile gradient design strategy for the fabrication of arbitrary objects using g-DLP. Step 4: Utilization of machine learning-driven multi-objective structural optimization to enhance two physical properties, reduce strain concentrations, and improve effective stiffness. Step 5: Optimization of gradient materials aimed at reducing stress concentrations and reinforcing structural integrity [78].

Related ML approaches have been used for lattice and gradient structures across multiple photopolymer materials. Peloquin et al. built a kernel ridge regression model that predicts key mechanical properties of 3D-printed photopolymer lattice structures (e.g., stiffness and strength) from structural porosity metrics and base material properties, showing accuracy comparable to finite element simulations but at a fraction of the computational effort [77]. Their study is based on a library of lattice designs spanning different porosity levels and base resins, with performance assessed on held-out geometries; however, the error is mainly reported as relative deviation from FE predictions. Nam et al. combined grayscale DLP with a machine learning-driven, multi-objective Bayesian optimization scheme to design polyurethane-acrylate gradient materials with tunable modulus and enhanced toughness [78]. The overview of their study is presented in Figure 6, where five steps from the development of highly tunable PUA to the optimization of gradient materials are shown with the assistance of machine learning. Their surrogate, based on Gaussian-process regression and sequential model-based optimization, identifies gradient profiles that reduce strain concentrations and delay crack initiation, directly targeting mechanical robustness rather than only geometric fidelity.

### 3.3. Machine Learning-Based Prediction of Mechanical Properties in Selective Laser Sintering

For polymer powder bed fusion, machine learning has mainly been used to capture how build layout and process parameters influence tensile behavior, and to speed up mechanical characterization. Most available studies focus on nylon-based powders and explore both the “forward” prediction of strength/modulus from process settings and hybrid schemes that combine simulation with data-driven models.

**Figure 7:**
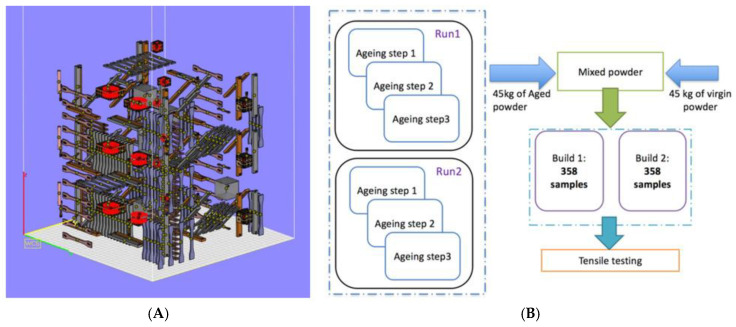
(**A**) Build layout in Magics 20.0. (**B**) Schematic representation of main stages of the experiment [79].

Baturynska developed one of the earliest SLS-oriented frameworks by training nonlinear ML models to predict the tensile modulus, nominal stress and elongation at break of nylon specimens from part placement, build orientation and simple STL-derived geometric descriptors (number of facets, surface area, volume) [79]. Figure 7A presents the build layout used and Figure 7B the main stages of the experiments. Using an EOS P395 system, 217 tensile specimens were fabricated in two identical builds (434 specimens in total), and models were trained both per orientation and on the pooled dataset. The research showed that nonlinear models (e.g., tree-based and kernel methods) significantly outperformed linear regression, often achieving >80% prediction accuracy despite the absence of explicit laser/process parameters, underlining that build layout alone already encodes a strong mechanical signature.

More recently, Tu et al. explicitly modeled the mapping between SLS process parameters and mechanical properties of nylon-12 parts using several data-driven approaches within both direct and inverse design frameworks [80]. In the direct mode, laser power, scan speed, hatch spacing and vertical position were used as inputs to estimate ultimate tensile strength, Young’s modulus and elongation at break; in the inverse mode, the desired mechanical properties were fed to the model to recommend suitable parameter combinations. The experimental campaign used 81 tested combinations of process parameters (each repeated three times), of which 74 unique data points were retained after excluding faulty tests. A 70/30 train–validation split was then applied, so that each second sample formed the validation set. Fuzzy inference systems (FIS), artificial neural networks (ANNs) and adaptive neuro-fuzzy inference systems (ANFIS) all achieved low prediction errors for the direct problem, with FIS slightly outperforming the others, while ANN was more accurate in the inverse problem, demonstrating that ML can effectively support both process optimization and property-driven parameter selection in SLS. The reported mean percentage error is approximately 6.2% for predicting ultimate tensile strength using FIS. Using the ANN approach, the average estimation error is about 5.21% for UTS, while the ANFIS method yields an error of roughly 3.3%.

Malashin et al. combined finite element modeling and ML to predict the tensile behavior of SLS-fabricated PA2200 components printed in different orientations [81]. Experimental tensile tests were used to calibrate FEM simulations, and then several regression algorithms (including k-nearest neighbors and support vector regression) were trained to forecast the tensile strength and especially relative elongation from geometric and orientation-related features. The best-performing models reproduced elongation with high accuracy, illustrating the benefit of coupling physics-based simulations with data-driven regressors for SLS mechanical design. However, the paper primarily reports MSE in physical units.

### 3.4. Machine Learning-Based Prediction of Mechanical Properties in Jetting Processes (Material and Binder)

**Figure 8:**
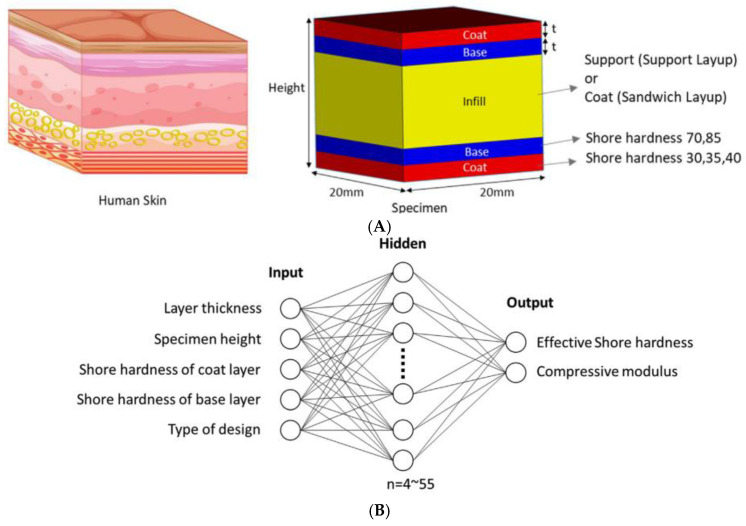
(**A**) Design of multi-material PolyJet-printed specimens taking inspiration from human skin. (**B**) Neural network architecture with 1 hidden layer for multi-objective optimization of shore hardness and compressive modulus [82].

In material jetting, Goh et al. [82] trained supervised machine learning models to predict the bulk mechanical response (compressive modulus and Shore hardness) of PolyJet-printed, multi-material tissue-mimicking anatomical models as a function of the internal layout of rigid and soft photopolymers (Figure 8A). They demonstrated that a feed-forward neural network (Figure 8B) can accurately approximate the mapping from designed material distribution to effective stiffness, enabling the inverse design of phantoms with target modulus ranges relevant to soft tissues. In their study, 216 specimens corresponding to 72 combinations of layer-stacking and material parameters were printed and tested, and the optimal 5–33–2 network architecture achieved a mean square error of about 1% for compressive modulus, outperforming a response surface model.

For binder jetting, most ML efforts have focused on metal or sand systems rather than polymers, but they illustrate how process–structure–property relationships can be captured and exploited [83,84]. Qian et al. [83] considered binder-jetted 2024Al and used an artificial neural network to predict the relative density, dimensional change and compressive strength of green parts from a dataset with different printing conditions (layer thickness, resolution and binder saturation); the ANN reached coefficients of determination above 0.92. Del Giudice et al. [84] studied sand-based binder jetting using a Latin Hypercube sampling of 18 print jobs and built polynomial-chaos surrogate models of compressive and flexural strength and Young’s modulus; the surrogates were validated through leave-one-out cross-validation and used for sensitivity analysis rather than pure accuracy maximization.

## 4. Discussion, Limitations, Future Trends

The previous section reviewed in detail how different groups have used ML models to relate process parameters to mechanical properties across the main polymer AM technologies. This section discusses trends that emerge from this body of work, the limitations that currently constrain the field, and possible future directions.

### 4.1. Technology-Dependent Trends

**Figure 9:**
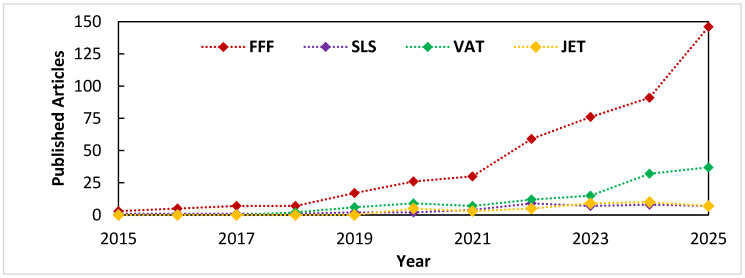
Number of journal articles (2015–2025) on the use of machine learning in polymer additive manufacturing, grouped by printing technology (FFF/FDM, SLS, vat photopolymerization, jetting). Data were obtained from a structured search in Scopus.

The bibliometric analysis in Figure 9 shows that FFF/FDM clearly dominates the literature on ML-based polymer additive manufacturing. The number of published articles that combine FFF with machine learning has grown almost exponentially since about 2020, reaching well over an order of magnitude more papers than any other polymer AM technology in 2025. VAT photopolymerization appears as the second most studied process, while SLS and jetting (both material and binder) remain comparatively underexplored.

This dominance of FFF/FDM is unsurprising. First, extrusion printers are inexpensive, widely available in both research labs and makerspaces, and are often open-hardware/open-software platforms. This lowers the barrier for generating sizeable experimental datasets. Second, mechanical performance in FFF is strongly and visibly affected by process parameters (layer height, infill density, nozzle temperature, etc.), so there is a clear incentive to develop predictive models that help tune these parameters. Third, because FFF parts are commonly used for prototyping and low-volume functional components, engineers are willing to iterate over many builds to calibrate ML models. In contrast, SLS and industrial VAT or jetting systems are more expensive and less accessible, rely on proprietary control software, and are frequently used in industrial settings where experimental time is costly and data cannot easily be shared, which slows down open academic work.

The differences in trends between technologies also reflect how naturally each process lends itself to data collection. For FFF, the number of controllable parameters is moderate and the time per build can be short, which enables relatively straightforward design-of-experiments and ML training. VAT processes share some of these advantages, but they additionally involve complex resin chemistry and curing kinetics, so experiments must be carefully controlled. For SLS and jetting, the process windows are high-dimensional and the underlying physics (powder spreading, heat transfer, binder infiltration and sintering) is more complex; this both increases the need for ML and makes it harder to generate clean, well-labeled datasets. As Figure 1 suggests, these practical considerations have so far translated into an uneven distribution of research effort, which should be kept in mind when comparing the apparent “maturity” of ML models across technologies.

### 4.2. Machine Learning Versus Classical Statistical Approaches

Historically, studies on process–property relationships in polymer AM have relied mainly on conventional statistical techniques such as ANOVA, Taguchi designs and response surface methodology (RSM). These approaches are still highly valuable because they are relatively simple, require modest data volumes and provide clear information on the main effects and low-order interactions of process parameters. Many of the machine learning studies discussed in Section 3 first employ Taguchi or RSM-based designs to generate a structured experimental dataset, and only then fit more flexible predictive models.

Modern ML methods, however, extend the capabilities of these classical tools in several ways. Neural networks, ensemble tree algorithms and support vector regression can represent strongly nonlinear trends and complex interactions among parameters, for example, the combined influence of the raster angle, layer thickness and temperature, far beyond what can be captured with low-order polynomial models. They also naturally support multi-output prediction, allowing tensile strength, modulus and elongation at break to be estimated simultaneously, and they can be embedded into inverse design frameworks where the desired properties are specified and the corresponding process windows are identified automatically. Furthermore, ML algorithms can incorporate heterogeneous inputs such as images from in situ monitoring, micro-CT-derived descriptors or features generated by simulations, something that traditional statistical models are not designed to handle.

### 4.3. Data Generation, Experimental Throughput and the Role of Simulation

A key limitation in training reliable ML models is the lack of sufficiently large and high quality datasets. Generating each data point typically involves printing specimens, performing mechanical testing and processing the resulting measurements. In addition, the available data in the literature are often obtained under different printing protocols, geometries and testing standards, which makes it difficult to merge datasets from different studies into a single coherent pool.

**Figure 10:**
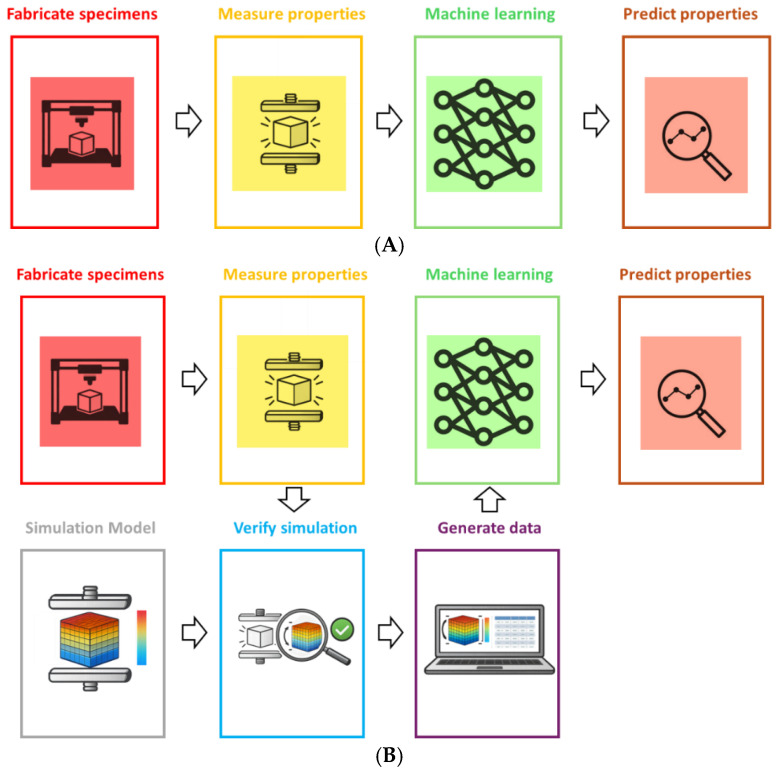
Schematic comparison of workflows for building machine learning models that predict mechanical properties from process parameters in polymer additive manufacturing. (**A**) illustrates the conventional, experiment-only pipeline: fabricate specimens, measure mechanical properties, train an ML model, and use it for property prediction. (**B**) shows a hybrid strategy in which a physics-based simulation (e.g., FEM) is first built and verified using a limited set of experiments and then used to generate large synthetic datasets that complement the measured data, enabling more robust and data-efficient ML models.

Figure 10 illustrates two general strategies that can be followed when building ML models. The upper workflow (Figure 10A) corresponds to the approach adopted in most existing publications: specimens are fabricated, their mechanical properties are measured, an ML model is trained on these measurements, and the trained model is then used to predict properties for new combinations of process parameters. In this purely experimental pipeline, the accuracy and generality of the model are strongly limited by the number and diversity of physically tested samples.

The lower workflow (Figure 10B) introduces an additional simulation step, typically based on finite element modeling or multiscale micromechanical models, in order to enrich the experimental data. In this hybrid approach, a relatively small but carefully designed experimental campaign is first used to calibrate and validate a numerical model. Once this model is considered reliable, it can be used to generate extensive synthetic datasets over a wide range of process conditions and geometries. These simulated data, possibly combined with the original experimental results in a multi fidelity setting, are then used to train the ML model. This strategy offers one large advantage: a limited set of experiments can be expanded into thousands of virtual samples and simulations can explore extreme regions of the process window that would be difficult or unsafe to test experimentally.

The main drawbacks of this hybrid route are the effort required to build and validate accurate simulations and the danger that any systematic error in the numerical model will be transferred into the ML predictions. Even so, ongoing improvements in computational power and multiscale modeling tools suggest that simulation informed or digital twin workflows will become increasingly important for data generation and ML training in polymer additive manufacturing.

### 4.4. Gaps in Mechanical Property Coverage

Another limitation highlighted by the literature survey is the strong emphasis on a very narrow set of mechanical properties. Most ML studies concentrate on quasi-static tensile strength and Young’s modulus, with a smaller number of works addressing flexural strength and hardness. In contrast, several property classes that are crucial for structural integrity and functional performance remain largely unexplored from a machine learning point of view.

Impact behavior and fracture toughness are rarely considered, even though many polymer AM components are subjected to dynamic loads. Charpy and Izod impact strength are rarely modeled as functions of process parameters. Similarly, only a few preliminary attempts have been made to predict fatigue life, S–N curves or long-term creep compliance in printed polymers, despite the fact that these time-dependent phenomena govern durability in service. Environmental and aging effects are also almost completely absent from current ML frameworks. The influence of humidity, ultraviolet exposure, thermal cycling or aggressive chemical environments is generally ignored, and most models implicitly assume them as printed specimens tested at room temperature.

For time- or cycle-dependent responses, ML models could predict the parameters of classical laws (for example fatigue curves or creep-viscoelastic models) from process parameters. Environmental factors such as temperature, humidity, UV dose or chemical exposure can be included as additional inputs. The main obstacles are data scarcity, the cost and duration of long-term tests, and the need to respect physical constraints.

Another gap concerns multiaxial and anisotropic behavior. Many existing models are calibrated for a single loading direction, whereas the layered architecture of additively manufactured parts leads to pronounced anisotropy. For robust structural design, a full constitutive description that includes shear and off axis loading is required, yet ML models of this type are still rare.

One final limitation is the poor standardization of how mechanical properties are produced and reported. The studies reviewed use different specimen geometries, internal architectures, ASTM/ISO standards, and loading or environmental conditions, which makes comparisons across datasets difficult and limits cross-study benchmarking and data reuse in ML. To make future datasets more suitable for ML, it would be useful to systematically identify the features that most strongly influence prediction. Based on such analyses, a common metadata template could be defined that at minimum records the test standard, complete specimen geometry and build orientation, all relevant process parameters, loading and environmental conditions, and, where available, full stress–strain curves in addition to scalar properties. Adopting this level of description would greatly improve the transferability and robustness of the trained models.

### 4.5. Future Trends

Looking ahead, several directions seem particularly promising for advancing ML in polymer additive manufacturing. A key one is the development of physics-informed and more interpretable models. By embedding basic physical constraints, ML predictions can become more robust and trustworthy. Closer integration with in situ monitoring is also expected, where ML uses both process parameters and live sensor data to predict final properties and eventually enable closed-loop control during printing.

## 5. Conclusions

This review summarized how machine learning is used to predict the mechanical properties of additively manufactured polymer parts from process parameters across extrusion, vat photopolymerization, SLS and jetting processes.

FDM and FFF clearly dominate the literature, where infill density, raster or build orientation and, for composites, fiber content emerge as the most influential variables for tensile and flexural behavior. Well-tuned neural networks and ensemble models routinely reach errors below about 5–10 percent for strength and modulus, showing that ML surrogates can substantially reduce trial and error in process setup, with tree-based ensembles offering additional interpretability. For DIW, the existing work confirms that ML can learn the coupling between ink rheology, toolpath and compressive response, although systematic datasets that link DIW parameters to standardized mechanical tests are still rare.

In vat photopolymerization, ML has been applied to bulk specimens, architected lattices and graded materials, indicating that it can efficiently explore the combined space of curing conditions, architecture and mechanical performance. For polymer SLS, models have been built to connect build layout and process settings to tensile properties, often in combination with finite element or molecular simulations, but studies remain strongly focused on PA12 and relatively small datasets. Machine learning for mechanical prediction in jetting processes is still in its infancy, with only a few works on PolyJet and almost none on polymer binder jetting.

Across all technologies, the main challenges are limited and fragmented datasets, a narrow focus on a few quasi-static properties, and the predominance of black box models tailored to specific material–printer combinations. Future progress will depend on more systematic data generation, including simulation-informed workflows, on broader property coverage that includes fatigue, impact and environmental effects, and on the development of physics-aware ML approaches.

## Data Availability

No new data were created or analyzed in this study.
